# Underwater Communications for Video Surveillance Systems at 2.4 GHz

**DOI:** 10.3390/s16101769

**Published:** 2016-10-23

**Authors:** Sandra Sendra, Jaime Lloret, Jose Miguel Jimenez, Joel J.P.C. Rodrigues

**Affiliations:** 1 Signal Theory, Telematics and Communications Department (TSTC), Universidad de Granada, C/Periodista Daniel Saucedo Aranda, s/n., Granada 18071, Spain; ssendra@ugr.es; 2 Integrated Management Coastal Research Institute, Universidad Politécnica de Valencia, C/Paranimf, n° 1, Grao de Gandia 46730, Spain; jojiher@dcom.upv.es; 3 National Institute of Telecommunications (Inatel), Santa Rita do Sapucaí 37540-000, Brazil; joeljr@ieee.org; 4 Instituto de Telecomunicações, Universidade da Beira Interior, Rua Marquês d'Ávila e Bolama, Covilhã 6201-001, Portugal; 5 ITMO University, 49 Kronverksky Pr., St. Petersburg 197101, Russia

**Keywords:** video transmission, underwater video surveillance, underwater communications, underwater wireless sensor network (UWSN), 2.4 GHz, freshwater, electromagnetic waves, modulations

## Abstract

Video surveillance is needed to control many activities performed in underwater environments. The use of wired media can be a problem since the material specially designed for underwater environments is very expensive. In order to transmit the images and videos wirelessly under water, three main technologies can be used: acoustic waves, which do not provide high bandwidth, optical signals, although the effect of light dispersion in water severely penalizes the transmitted signals and therefore, despite offering high transfer rates, the maximum distance is very small, and electromagnetic (EM) waves, which can provide enough bandwidth for video delivery. In the cases where the distance between transmitter and receiver is short, the use of EM waves would be an interesting option since they provide high enough data transfer rates to transmit videos with high resolution. This paper presents a practical study of the behavior of EM waves at 2.4 GHz in freshwater underwater environments. First, we discuss the minimum requirements of a network to allow video delivery. From these results, we measure the maximum distance between nodes and the round trip time (RTT) value depending on several parameters such as data transfer rate, signal modulations, working frequency, and water temperature. The results are statistically analyzed to determine their relation. Finally, the EM waves’ behavior is modeled by a set of equations. The results show that there are some combinations of working frequency, modulation, transfer rate and temperature that offer better results than others. Our work shows that short communication distances with high data transfer rates is feasible.

## 1. Introduction

Over the past 20 years, human activity in aquatic and marine environments has increased. Moreover, the development and application of aquaculture technology has led the creation of new research lines. Consequently, the impact on marine ecosystems has also increased. Human activity generates unwanted wastes in rock reefs, benthic habitats or sensitive sea grass communities, mainly from the food not consumed by the fish and the faeces and waste thereof. In addition, due to food waste and the consequent economic impact, fish farming is often not considered a feasible activity.

In recent years, research activity in underwater wireless communications and underwater ad hoc networks has experienced an enormous increase. Most experts agree that the future of the sector needs some improvements in the competitiveness of enterprises to be profitable in the global market, through the incorporation of new technologies. Nowadays, some researchers are developing low-cost sensors with very good features. However, underwater wireless communication remains a challenge, especially when the amount of information sent between devices requires high bandwidth, like video.

Current research activity is focused on increasing the distance between devices and increasing the bandwidth. The research community is also trying to increase the network lifetime by reducing the energy consumption of underwater devices [1,2]. In order to work with underwater wireless systems, we should know and understand how waves can propagate through the water environment. Because of the physicochemical characteristics of water [3] and the constraints of the medium [4,5], the technology used to propagate the communication will present a set of benefits and drawbacks. In addition, the EM wave’s propagation through water is very different from the propagation through air because of water's high permittivity and electrical conductivity. The wave’s attenuation is high in the water, compared to the air, and increases rapidly as a function of the frequency. With a relative permittivity of 80, water is one of the materials with the highest permittivity and this has a significant impact on the angle of refraction at the air-water interface.

In order to implement an underwater wireless sensor network (UWSN), researchers usually use acoustic waves. This kind of waves presents better behavior in turbid waters and they are less susceptible to being affected by particles suspended in the water. It is the most used method because acoustic waves can reach distances of up to 20 km [6]. Acoustic communication is a very well proven technology. However, it presents several drawbacks such as low data rate (up to 20,000 bps approximately) which is mainly limited by several parameters like strong reflections and low carrier frequency that generates an important attenuation when the communication is carried out in shallow waters [7]. Other parameters such as salinity or temperature also worsen the communication performance. To mitigate these effects and achieve higher data rates, researchers usually work with different modulation and communication protocols.

We can also find underwater communication devices based on optical systems. These devices present very high propagation speed, but they are affected by the water turbidity and register a strong backscattering phenomenon due to particles suspended in the water. For this reason, optical signals are not good options for long distances.

Finally, EM waves, working in the radio frequency (RF) range, can also be considered as a good option to implement underwater wireless communication systems. EM waves can achieve data transfer rates of up to 100 Mbps for very short distances. They also present substantial immunity to several environmental features. EM waves are less affected than acoustic waves by the refraction and reflection effects in water with little depth. In addition, suspended particles have very little effect on them. The speed of EM waves is around 150,000 times higher than that of acoustic ones, and it depends on the conductivity (σ), permeability (μ), volume charge density (ρ) and permittivity (ε) of the medium [8]. It is important to know that these parameters usually change depending on the type of water and they are associated with the electrical conductivity value. Seawater and freshwater have different conductivity values. Drinking water can have conductivity values ranging from 0.005–0.05 S/m for, while seawater can have values up to 4 S/m. The conductivity of fresh water presents a typical value of 0.01 S/m. However, the permittivity value of seawater is directly related to changes in salinity, temperature and working frequency.

Regarding EM wave propagation, it is hampered by the high attenuation due to the water conductivity. The EM wave propagation speed and absorption coefficient are directly related to the working frequency. If the working frequency increases, the signal attenuation also increases. Hence, the higher frequencies will register greater signal losses [9,10,11].

Most of the studies analyze losses and signal attenuation as a function of working frequency. However, there are few studies that provide analysis considering modulation type, data transfer rates and the water characteristics [12].

Because of the limited information about underwater communication based on EM waves and the possibility of using them in many applications, this paper presents a practical study to demonstrate that EM waves are the best option to transmit video through underwater environments. To do this, firstly, we have analyzed the minimum network requirements to transmit a video with a specific format. After that, underwater measurements are performed taking into account all modulation schemes defined in the IEEE 802.11 standard [13] for the 2.4 GHz Industrial, Scientific and Medical (ISM) frequency band. Measurements are carried out in fresh water. In [14], the authors compared the signal transmission in underwater environments for 700 MHz, 2.4 GHz and 5 GHz frequencies. As we noted, when the 5 GHz frequency band is used, the results are bad (less than 12 cm). However, for 700 MHz, the maximum distance achieved is significantly larger (around 200 cm). Therefore, we know that the 915 MHz frequency will achieve larger distances than 2.4 GHz. On the other hand, its available bandwidth for video transmission will decrease considerably. The reason for using devices compatible with the IEEE 802.11 standard is because they are cheap and implement a modulator and demodulator for this frequency. The study shows the network performance for different water conditions when the water temperature is changed. The tests are carried out for different configurations. The modified parameters are the data transfer rates, signal modulation and working frequency. The current paper improves our previous works [15,16] by providing a more detailed study which includes higher data bit rates, other coding techniques and an analysis of performance depending on the temperature. These new results have shown lower round trip times (RTTs) and larger distances. In order to determine if the analyzed parameters present a relationship with distance and between them, we analyze the results obtained from each case by using the analysis of variance (ANOVA). We use a two-way ANOVA. Finally, we will use these results to estimate the analytical models that describe the operation and behavior of signals in underwater environments, taking into account all analyzed parameters.

The rest of the paper is structured as follows: Section 2 reviews some works and studies where researchers analyze the performance of EM waves when working in fresh water. Section 3 explains the preliminary tests to know the minimum requirements needed for an underwater video transmission. It is also presented the scenario where underwater tests have been performed as well as the equipment and the software used in our tests. The section includes a description of the different tests, the parameters changed to perform our measurement and the statistical tools used to define the relationship between parameters. The results of our measurements and Analysis of Variance (ANOVA) values related to these results are shown in Section 4. Section 5 presents the values of the average RTT measured for different values of temperature, modulation and working frequency, for maximum distances. Section 6 shows the mathematical expressions that model the behavior of the signal depending on the water temperature, modulation and working frequency. Finally, Section 7 shows the conclusion and future work.

## 2. Related Work

In order to implement an underwater ad hoc wireless network for video transmission, it is important to know the minimum bandwidth for video transmission. In the related literature, we can find some proposals where video is sent over wireless technologies such as IEEE 802.11n Standard [17]. In addition, knowing the best way of compressing and processing video is also important [18,19]. On the one hand, there are some works where authors analyze underwater wireless communications. On the other hand, there are very few papers about underwater communications based on EM waves. However, it is easy find to several proposals about underwater acoustic communication. This section provides a concise and precise description of the experimental results and their interpretation as well as their conclusions.

Che et al. [20] investigated the possibility of designing and implementing a marine wireless sensor network based on RF EM communications. The authors developed a small scale wireless sensor network (WSN) to collect data from the seabed and monitor the effects of underwater coastal erosion. Their proposal was based on a multi-hop WSN with a fixed topology of 30 nodes. Ad hoc On-Demand Distance Vector Routing (AODV) was used as routing protocol. The system used low frequency in order to achieve a 40 m transmission radius. However, the data transfer rate is only 100 bps. The authors concluded that this network needed to implement some failure tolerance mechanism to maintain a certain confidence level before a manual intervention. They also explained that when a node failed, the network suffered a worse convergence time, although the performance remained in acceptable level due to the features of the AODV protocol.

A RF system was presented in [21] by Shaw et al. as a possible solution to transmit information with higher transmission data rates in underwater environments. The transmitter used tank tests with a direct digital synthesizer (DDS) and a PIC microcontroller in charge of processing the information. The DDS worked between 0.5 MHz to 25 MHz. As the results showed, in sea water, RF signals at frequencies up to 5 MHz can be transmitted over a distance of 90 m. However, the results for a bigger salt concentration showed that the attenuation increased with distance, with a rapid attenuation for low distances and a more gradual progression for longer distances.

Joe and Toh presented in [22] electric current method based on a pair of copper plate electrodes used as antennae for implementing a digital underwater communication system with high data rates and short range. The experiment was carried out in a fiber glass aquarium with sea water and seabed sand. The experimental results showed that this system is able of transmitting data with higher bit rates over short ranges compared to the acoustic methods.

Goh et al. presented in [23] some recent investigations that demonstrated it is possible to use EM waves up to the MHz frequency range to implement wireless links in seawater. The results showed that EM wave propagation in sea water is highly affected by parameters such as permittivity and conductivity. The authors also showed that the EM wave propagation presented high attenuation in the near field. However, the transmission in the far field region can allow transmissions over longer distances and applications such as compressed video, radar or data telemetry could use this system.

Cella et al. [24] investigated underwater wireless links in a shallow water environment from two points of view, i.e., experimental and theoretical. Authors described a model useful to describe the shallow water environment from an EM point of view. From these analyses, authors defined the maximum distance coverable with a node transmitting with a specific power. The tests were performed with an electric dipole in a homogeneous and a stratified dielectric. The results showed that lateral wave propagation can be used for wireless communication in shallow water environment.

Finally, some authors of this paper have reported several previous works about underwater communications based on radio frequency (RF). In all cases, the authors used the 2.4 GHz ISM frequency band. On the one hand, Sendra et al. carried out different practical tests where parameters such as frequency and signal modulation were changed to check several the minimum depth at which devices should be placed, distance between devices and the best combination of transmission parameters [15,16]. This work only measured the number of lost packets and RTT for 1, 2, 5.5 and 11 Mbps from 2.412 to 2.442 GHz. These tests were performed at 26 °C and the maximum achieved distance between devices was 17 cm. Authors provided some experimental models from the communication tests performed in fresh water using EM waves in the 2.4 GHz ISM frequency band. In addition, the authors compared their communication system proposal with existing systems. They concluded that Binary phase shift keying (BPSK) and Quadrature Phase-Shift Keying (QPSK) presented better performance than the other analyzed modulations. Furthermore, the system presented better results at 2.432 GHz than when working at 2.412 GHz.

As far as we know, there are no studies on underwater communications based on EM waves where the authors analyze the communication from the point of view of the temperature of the medium, modulation and data transfer rates and their effects on performance and maximum distance.

## 3. Scenario and Test Bench

The measurements have been performed in fresh water under specific conditions in a controlled space. This section present the scenario where tests have been performed and the equipment and the software used in our tests. Finally, we will explain the different tests, the parameters changed to perform our measurement and the statistical tools used to define the relationships between parameters.

### 3.1. Preliminary Tests to Characterize the Video Transmission

In order to specify the minimum requirements of the communication channel, the first step is to define the requirements that the file we want to transmit needs, so have done a preliminary test where a SJCAM5000 WiFi action camera [25] has been used. It is a small camera capable of recording images in full HD quality and transmits its signal via Wi-Fi. The SJCAM 5000WIFI uses the optical sensor MN34110PA developed by Panasonic Corporation [26] with a photograph resolution of 14 Mpx. This camera usually works with the following resolutions: 1080 p at 30 fps and 720 p at 60 fps. During the tests, we have used many video resolutions in order to make this study useful for many other cameras.

As file to be sent, we have used a video of 50 s long compressed with the H.264 codec, which gives as a result an mp4 file format. We have used various resolutions as well as two frame frequency commonly used in this type of cameras. Table 1 summarizes the configuration parameters for the video used in this test.

**Figure 1:**
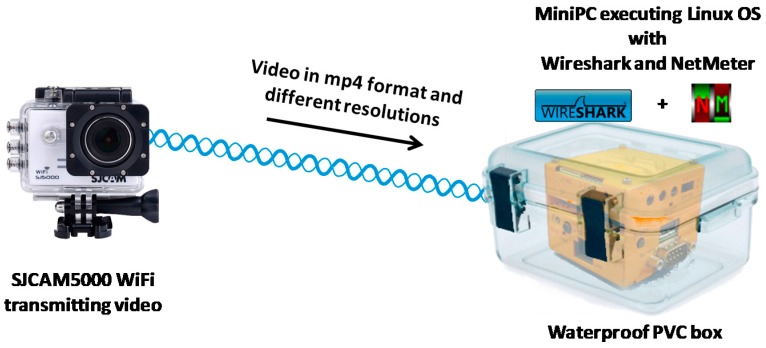
Test bench for video characterization.

To perform these tests we have used the action camera and a miniPC executing a lightweight OS based on linux. This kind of device is able to work as a personal computer with the same functions, but its size is around a cube of 10 × 10 × 10 cm^3^ this device will serve us as a gathering system [27]. Both devices are connected through a point-to-point wireless connection. The camera records video and send it to the miniPC which monitors the network activity (see Figure 1). To register network activity, the miniPC runs Wireshark Network Analyzer [28] and NetMeter [29].

**Figure 2:**
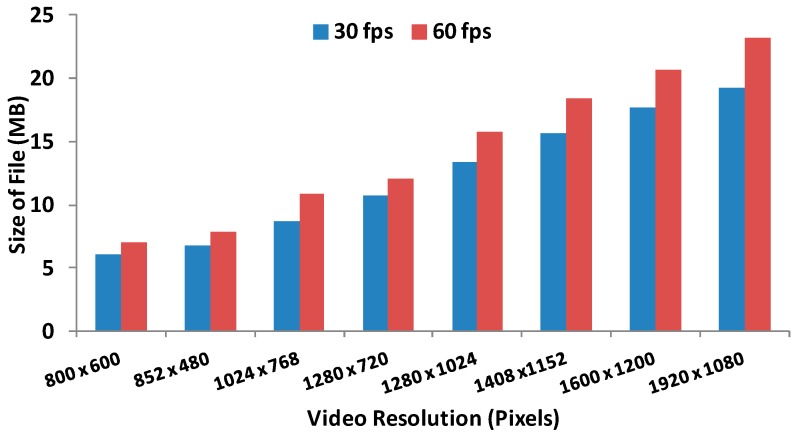
Size of files in MB.

The first parameter to be measured is the size of file we send through the network as a function of the resolution and the frame frequency. Figure 2 shows the size of file sent through the network in MB.

**Figure 3:**
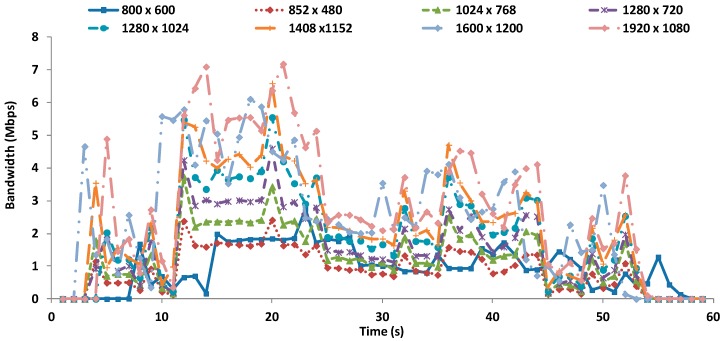
Bandwidth for videos at 30 fps.

The most noteworthy aspect is that for a given resolution, having a double frame frequency does not mean having a twice bigger file. Figure 3 shows the bandwidth in Mbps as a function of time for each video resolution when they are transmitter at 30 fps. As we can see, the maximum bandwidth (7 Mbps) is registered for the biggest resolution (1920 × 1080).

**Figure 4:**
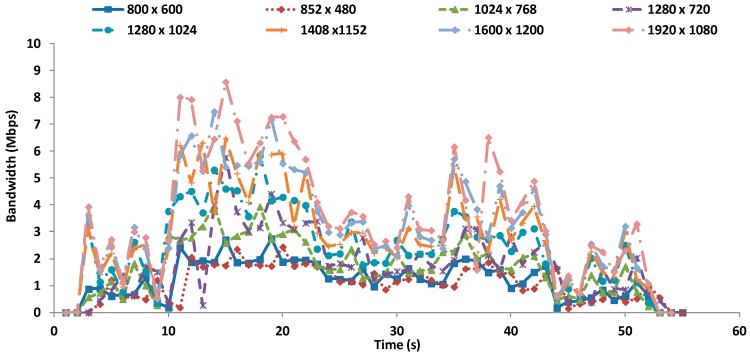
Bandwidth for videos at 60 fps.

Figure 4 shows the bandwidth in Mbps for each video resolution when they are transmitted at 60 fps. In this case, the maximum bandwidth is about 8.5 Mbps and it is obviously registered for the biggest resolution (1920 × 1080).

**Figure 5:**
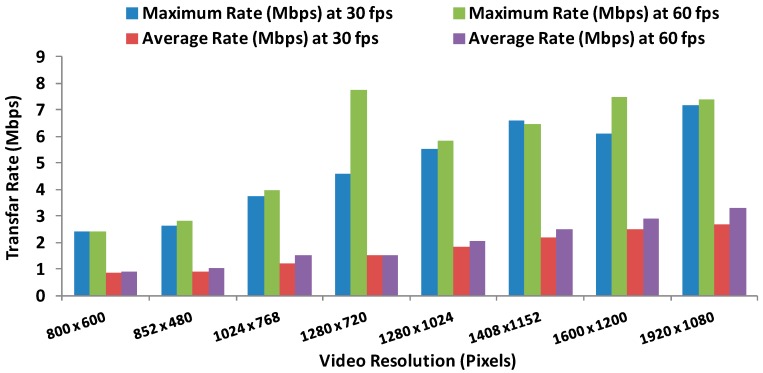
Maximum and average data transfer rate for each video at 30 fps and 60 fps.

Finally, Figure 5 shows the maximum and average data transfer rate for each video at 30 fps and 60 fps. If we are unable to guarantee these values, at certain times the video will be received with errors and will suffer cuts and interrupts.

As we have seen in previous studies [15], the acoustic waves do not guarantee enough bandwidth to transmit such content, so we need to increase it and for this reason, we want to study the behavior of EM waves. This is the method with low cost of implementation that permits the video transmission.

### 3.2. Scenario Description for Underwater Measurements 

The measurements are performed in a fresh water swimming pool. The chemical parameters of water have been measured. It contains 0.3 mg/L of dissolved chlorine and bromine and the water pH is 7.2. The swimming pool is 8 m length and 4 m wide with a depth that ranges between 1.5 m. and 1.80 m. The size of pool allows us avoiding any reflection on the walls, ground and water surface. The swimming pool has been made with bricks covered by small tiles of mosaic.

To carry out the tests, it is configured an ad hoc wireless connection between two laptops that have two vertical monopole antennae connected to their wireless cards. The laptops are maintained outside the water. We have used two HP pavilion dv6-6c13ss Intel Core i7 2670QM with 4GB RAM memory. The antennae, that have 2 dBi of gain, are placed inside the water. They are connected to each laptop using a 3 m pigtail.

The goal of these tests is the design of a system composed by an antenna and a small electronic board placed inside a watertight plastic box. Then, we evaluate if the antenna is able to transmit to the water when the medium changes. To do it, each antenna is put in a sealed plastic box (of 23.5 cm height, 8.7 cm width and 4.2 cm thickness) to make it watertight. The antennae are placed in the middle of the plastic box. Both antennae are faced by the widest part of the plastic box, so that the space between the antenna and the water is 2.1 cm. On the other hand, our system does not require the adaptation that a reviewer appoints because the antenna is surrounded by an air gap, as it is put inside a plastic and sealed box/container. For this reason the antenna starts to emit inside a small air gap created before the signal is introduced into the water. However and in order to be sure about this fact (because there is not previous work about it), we performed a preliminary test to ensure that the space between the antennae and the water does not influence over the maximum distance between devices. Because we are working with antennae prepared to transmit in air, we had to base our test in the wave’s behavior in the medium where our antennae started to transmit. To perform these tests, we have used commercial 2.4 GHz wireless devices with a power transmission 100 mW. In [30], we tested different values of power from 100 mW to 800 mW. We observed that the transmission behavior worsens. In fact, the maximum distance between devices did not increase due to probably to the big strength of reflections and refractions. For this reason, we finally decided to work at 100 mW. As we stated in one of our previous work [31], the theoretical transmitted power for an IEEE 802.11 WLAN device is –40.2 dBm at 1 m. This is the maximum power it is possible to reach. For this reason, we designed a container that allowed having 1 m of space between the antenna and the water.

**Figure 6:**
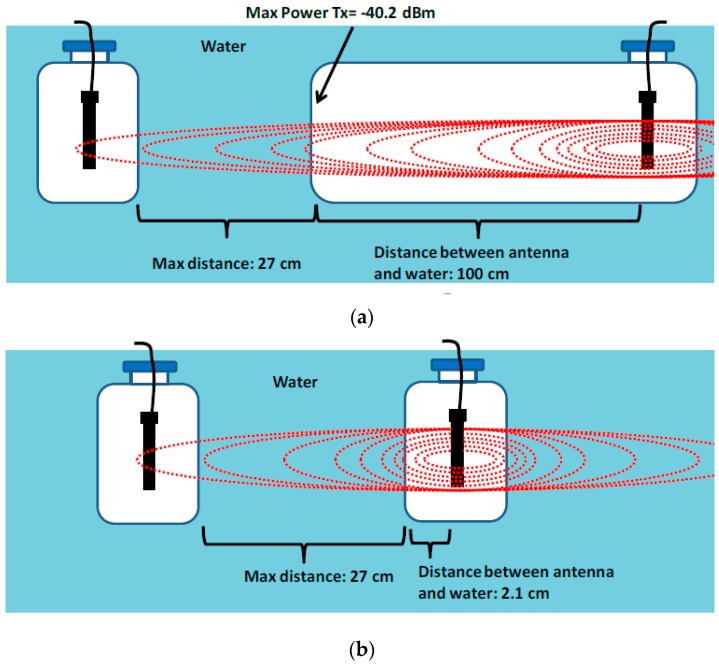
Preliminary test to check the effect of air gap. (**a**) distance between antenna and water: 100 cm; (**b**) distance between antenna and water: 2.1 cm.

Theoretically, we had to have longer distances because we allowed to the antenna to transmit with its maximum power transmission before transmitting through the water. However, the maximum distances obtained in both cases were the same, i.e., 27 cm (see Figure 6). For this reason, we finally decided to the use the small container. The goal of these tests is the design of a system, composed by an antenna and a small electronic board placed inside a watertight plastic box. Then, we evaluate if the antenna is able to transmit to the water when the medium changes.

**Figure 7:**
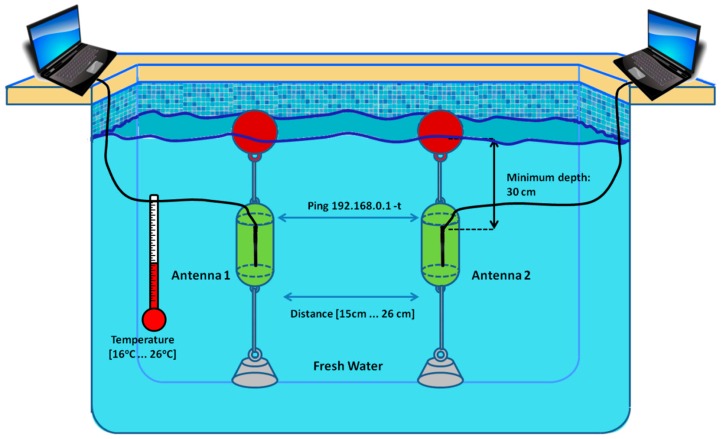
Swimming pool where measures have been taken.

As Figure 7 shows, both antennae should be placed under the water. They were placed at enough depth to avoid any transmission from the open air and at enough distance to the pool ground, walls and surface to avoid reflections.

When performing measurements in underwater environments, it is needed to ensure that signal does not spread out of the water. To guarantee this fact, we have proceeded to establish an ad hoc wireless connection between both antennae outside the water. After that, one of these antennae has been progressively introduced inside the water. The connection between both devices is checked every 5 cm until 30 cm. At this depth, the antenna located inside the water does not detect any signal from the antenna placed outside the water. This simple test helps us to ensure that measures are valid and the signal does not spread out of the water. From this moment, the second antenna can be put inside de water to start the tests.

### 3.3. Test Bench and Measurement Process

To perform the test bench and monitor the network activity, both computers run a MS-DOS shell. This tool offers us several network commands which allow us to see the network activity. In this case, we have used the ping command that provides the RTT for each packet. These tests analyze the values of RTT between both devices depending on several parameters. The parameters under study are the distance, modulation, working frequency and temperature. In addition, the ping command and the RTT will indicate us if the connection between devices is possible.

The average values of RTT are calculated considering only the packets that were successfully received. If a packet was not received or it was wrongly received, the value of RTT assigned is 3000 ms. This value was not taken into account in the RTT average calculation. It is very common the use of this value to define wrong/not received packets [32].

Tests were performed in the frequency range between 2412 MHz and 2462 MHz (specified by the IEEE 802.11b/g standard). Because of the effect of water and temperature, each temperature allows limited frequencies to perform our tests. Taking into account this fact, the tests for 16 °C were performed for the frequencies between 2412 MHz and 2452 MHz. At higher frequency values were not possible to establish an underwater wireless link. At 18 °C, we could take measurements for frequencies between 2412 MHz and 2452 MHz. We were also able to establish an underwater link for frequencies between 2457 MHz and 2462 MHz at 20 °C. At 22 °C, the highest frequency that allows establishing an underwater wireless link was 2452 MHz. Finally, Orthogonal Frequency-Division Multiple Access (OFDM) was measured for all temperatures, in exception of 26 °C because it did not work. At 26 °C, the frequency range analyzed was 2412 MHz to 2442 MHz. Table 2 summarizes the parameters analyzed in the performed tests.

### 3.4. Analysis of Variance by Two-Way (ANOVA)

The best way to check if there is any relationship between two parameters is analyzing the variance of these variables by an ANOVA.

Our analysis will compare the data transfer rate and the working frequency used at each temperature and modulation. The working frequency and the data transfer rates modulation are the groups to be compared. The variable under study is the maximum distance between antennae at each temperature.

As a summary, to perform this study, we have to take into account several things. Firstly, to avoid any bias during the measuring process, we have performed 100 sets of measurements with 30 repetitions per set. This kind of analysis requires several sets of measurements with several repetitions. The first step was checking if these sets of measurements followed a normal distribution. This step can be done using the descriptive analysis or checking if residuals follow the null hypothesis or the alternative hypothesis (as this section explains). From this moment, we can apply the ANOVA studies over the set of measurements [33]. For each ANOVA analysis, we consider two possibilities. This analysis compares individually the dependence of maximum distance between antennae and the data transfer rate (rows) and the dependence of the maximum distance between antennae and working frequency (columns). In both cases, the ANOVA poses a null hypothesis where there is non-dependence between parameters and an alternative hypothesis which express the dependence between them.

Analysis of rows:

We formulate two hypotheses:

- H0 = maximum distance does not depend on the data transfer rate (null hypothesis).
- H1 = maximum distance depends on the data transfer rate (alternative hypothesis).

Analysis of columns:

We formulate two hypotheses:

- H0 = maximum distance does not depend on the working frequency (null hypothesis).
- H1 = maximum distance depends on the working frequency (alternative hypothesis).

To define, in each case, whether the alternative hypothesis should be rejected or not, we take as important parameters the statistical value of F, the critical value of F, which is calculated from the value of significance level α. In our case, we have fixed our value as α = 0.05. The result of analysis of variances indicates the statistical value of “F” for each analyzed factor. In order to see if results present any significance (i.e., if the probability “P” has a value less than 0.05), the observed value of “F” needs to be, at least, equal to the critical value of F.

This will mean that there is a significant relationship between the analyzed factors (working frequency and data transfer rate) and our results (maximum distance between antennae). In that case, the results show that both parameters are statistically significant. If this comparison is not met, we would conclude that the relationship between parameters is not significant and its variations are due to the randomness of the measures. Before performing ANOVAs, we have analyzed if measurements follow a normal distribution. To perform this task, we have checked the descriptive statistic of each set of measurements. In concrete, the kurtosis and skewness coefficients are analyzed. The results gave us that the coefficients were between ±2, so it is possible to apply the ANOVA as a method of analysis of variance, because data follow a normal distribution.

In addition to see if groups of measurements follow a normal distribution, we can also see if they have some influence over the analyzed variable. To do it, we can calculate the residuals of each group. The residuals are calculated as the difference between the average value of each group and each value (see Table 3). From the results, the null hypothesis and alternative hypothesis can also be presented:

H0: *X_1_ = X_2_ = … = X_I_ = 0* ← Factor under study does not influence the parameter to be analyzed.
H1: any *X_I_ ≠ 0* ← Factor under study shows some influence over parameter to be analyzed.

where *X_I_* is the result of residuals calculation.

## 4. Results of Maximum Distances

This section shows the obtained results. Each subsection represents the maximum distance between the two antennae and the average RTT in ms. registered for each distance depending on the working frequency and the water temperature.

### 4.1. Measurements for BPSK at Different Temperatures

**Figure 8:**
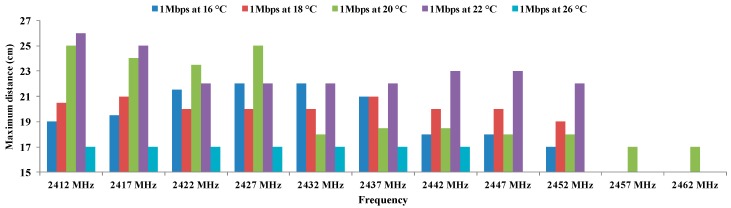
Maximum distances for BPSK.

Figure 8 shows the values of maximum distance for 1 Mbps using BPSK modulation for temperatures of 16, 18, 20, 22, and 26 °C.

It is important to highlight several relevant aspects. The first and the most significant is the variations obtained as a function of temperature. We can see that smaller distance values are obtained at 26 °C. Temperatures of 16 °C and 18 °C also present low values of distance, whereas for the rest of temperatures are recorded distances above 22 cm. Different working frequencies also offer different values of maximum distance. The longest distance is registered for frequency of 2424 MHz at 22 °C, followed by the frequencies 2412 MHz and 2427 MHz at 22 °C.

To analyze the relationship between the maximum distance, working frequency and the modulation as a function of the temperature, we use a two-way ANOVA where the working frequencies and temperature are the groups to be compared. The variable under study is the maximum distance between antennae when BPSK is used.

Table 4 shows the calculation of residuals, for BPSK modulation. We can observe that all values match with the hypothesis H1 where all residuals are different to 0. So, we are sure that these parameters cause some effect over maximum distance.

We can now focus our analysis on the dependence of maximum distance with working frequency and temperature. Table 5 shows the analysis of variance measurements at 16 °C. We see that in the case of rows, the probability value (0.00011845) is lower than the one defined for α. Therefore, the variation of results is significant. On the other hand, the value of F (7.60026754) is higher than the critical value of F (2.60597495). For this reason, we discard the null hypothesis and take the alternative hypothesis. This means that the maximum distance for each frequency depend on the temperature.

On the other hand, for columns, the value of the probability (1.9998 × 10^−9^) is lower than defined for α. Therefore, the variation of the results is also significant. Moreover, the value of F (12.4222303) is higher than the critical value of F (2.07724805). We can discard the null hypotheses for taking the alternative which means that the maximum distance between antennae using BPSK modulation depends on the working frequency.

These results show that there are significant differences between groups which are due to the increase in the working frequency and temperature and not due to the random effect. With this, we have checked that the maximum distance for BPSK modulation are statistically different and they depend significantly on the working frequency and temperature.

### 4.2. Measurements for QPSK at Different Temperatures

**Figure 9:**
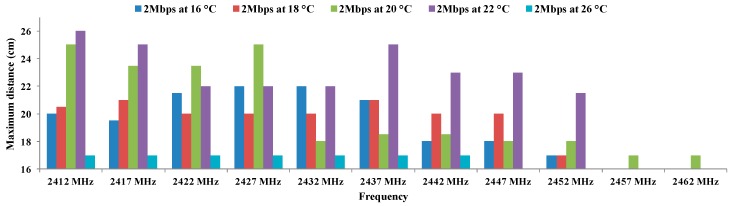
Maximum distances for QPSK.

Figure 9 shows the values of maximum distance for 2 Mbps which uses QPSK modulation at 16 °C, 18 °C, 20 °C, 22 °C, and 26 °C. This modulation also presents effects as a function of the temperature. The smaller distances are obtained for the temperature of 26 °C. Temperatures of 16 °C and 18 °C have maximum distances below 22 cm, while the frequencies that come from 2412 MHz to 2427 MHz at 20 °C achieve distances between 23 and 25 cm. Finally, we can see that the greatest distances are achieved for 2412 MHz at 22 °C.

The ANOVA for QPSK uses the frequency and each temperature as a group to be compared and in this case, the variable to be studied is the maximum distance between antennae using QPSK modulation. Table 6 shows the calculation of residuals, for QPSK modulation. We can observe that all values match with the hypothesis H1 where all residuals are different to 0. So, we are sure that these parameters cause some effect over maximum distance.

Table 7 shows de ANOVA results of dependency between QPSK modulation, working frequency and temperature. We see that in the case of rows, the probability value (0.00031136) is lower than the one defined for α. Therefore, the variation of results is significant. On the other hand, the value of F (6.71606994) is higher than the critical value of F (2.60597495). For this reason, we discard the null hypothesis and consider the alternative hypothesis. This means that the maximum distance for each frequency depend on the temperature.

On the other hand, for columns, the value of the probability (5.3453 × 10^−11^) is lower than defined for α. Therefore, the variation of the results is also significant. Moreover, the value of F (15.8849444) is higher than the critical value of F (2.07724805). We can discard the null hypotheses for taking the alternative which means that the maximum distance between antennae using QPSK modulation depends on the working frequency.

These results show that there are significant differences between groups which are due to the increase in the working frequency and temperature and not due to the random effect. With this, we have checked that the maximum distance for QPSK modulation are statistically different and they depend significantly on the working frequency and temperature.

### 4.3. Measurements for CCK at Different Temperatures

**Figure 10:**
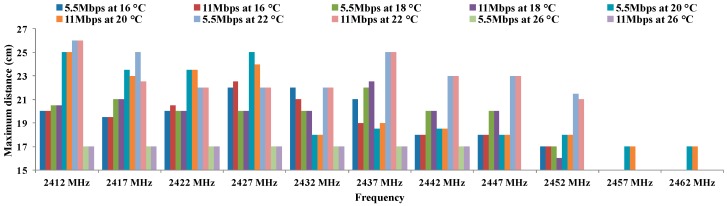
Maximum distances for CCK.

Figure 10 shows the values of maximum distance for the CCK coding scheme which has two values of data transfer rate (5.5 Mbps and 11 Mbps) at 16, 18, 20, 22 and 26 °C. The first point to note is that the largest distances are obtained in all cases for the temperatures of 20 °C and 22 °C. The best results are obtained at a frequency of 2412 MHz and 22 °C. Both data transfer rates achieve the same distance. Other values that can be considered good enough at 20 °C are 5.5 Mbps for 2427 MHz and 5.5 Mbps and 11 Mbps at 22 °C for 2437 MHz. The remaining results show lower distances between antennae.

Table 8 and Table 9 show the calculation of residuals, for CCK modulation at 5.5 Mbps and 11 Mbps. We can observe that all values match with the hypothesis H1 where all residuals are different to 0. So, we are sure that these parameters cause some effect over maximum distance.

Table 10 shows the results of ANOVA for CCK coding scheme for temperatures under study.

We see that in the case of rows, the probability value (2.0984 × 10^−8^) is lower than the one defined for α. Therefore, the variation of results is significant. On the other hand, the value of F (7.79263227) is higher than the critical value of F (1.98559496). For this reason, we discard the null hypothesis and take the alternative hypothesis. This means that the maximum distance for each frequency depends on the temperature.

On the other hand, for columns, the value of the probability (6.1007 × 10^−24^) is lower than defined for α. Therefore, the variation of the results is also significant. Moreover, the value of F (28.8675745) is higher than the critical value of F (1.93756679). We can discard the null hypotheses for taking the alternative which means that the maximum distance between antennae using CCK modulation depends on the working frequency.

These results show that there are significant differences between groups which are due to the increase in the working frequency and temperature and not due to a random effect. With this, we have checked that the maximum distance for CCK modulation are statistically different and they depend significantly on the working frequency and temperature. Finally, we have checked that the maximum distance is not affected by changes in data transfer rates.

### 4.4. Measurements for OFDM at Different Temperatures

**Figure 11:**
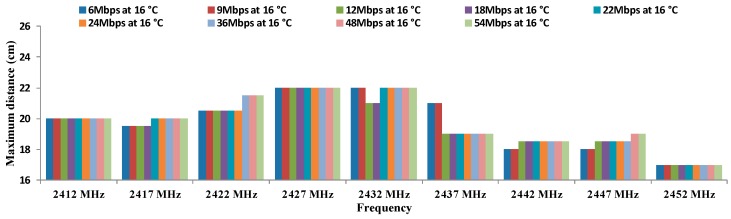
Maximum distances for OFDM at 16 °C.

Due to the large number of parameters to be represented, the OFDM results are grouped by temperature. Figure 11 shows the results of the maximum distances between antennae for all data transfer rates referred in OFDM modulation at 16 °C. As we can see, the maximum distance (22 cm) are registered for 2427 MHz and 2432 MHz for all data transfer rates. The lowest distances are registered at 2452 MHz.

**Figure 12:**
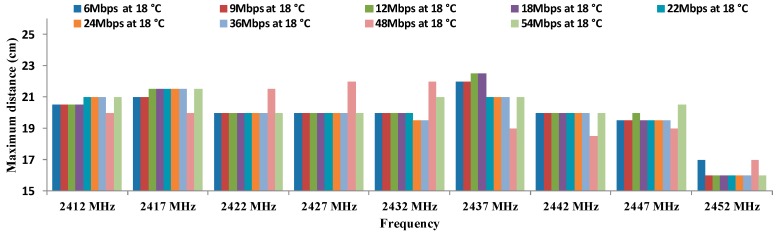
Maximum distances for OFDM at 18 °C.

Figure 12 shows the results of the maximum distances between antennae, for all data transfer rates in OFDM modulation at 18 °C. In this case, the maximum distance is registered for 12 Mbps and 18 Mbps at 2437 MHz. Again, the worst results (16–17 cm) are registered for highest frequency at all data transfer rates.

**Figure 13:**
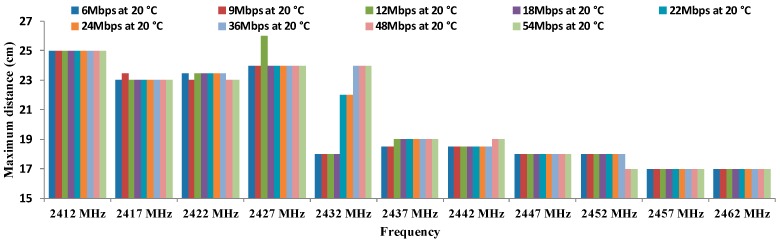
Maximum distances for OFDM at 20 °C.

Figure 13 shows the results of the maximum distance registered between both antennae depending on the working frequency for all data transfer rates considered in OFDM modulation at 20 °C. As Figure 12 shows the best results (25 cm) are registered for the lowest frequency (2412 MHz) for all data transfer rates and 12 MHz at 2427 MHz (26 cm). From 2437 MHz the maximum distance achieved is 19 cm.

**Figure 14:**
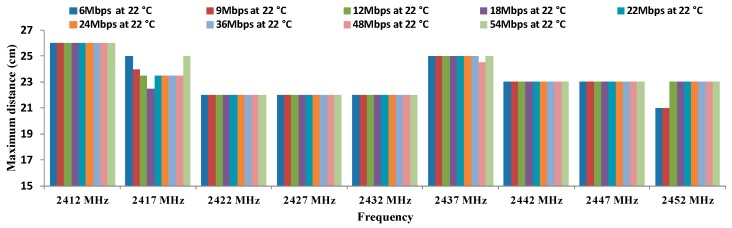
Maximum distances for OFDM at 22 °C.

Figure 14 shows the results of the maximum distance registered between both antennae depending on the as a function of working frequency for all data transfer rates considered in OFDM modulation at 22 °C. In this case, the maximum distance is registered for the lowest frequency (2412 MHz). In addition, it is important to highlight that the minimum distance for this temperature is 22 cm. However, we cannot use OFDM for temperatures higher than 22 °C.

Finally, Table 11, Table 12, Table 13, Table 14, Table 15, Table 16, Table 17, Table 18 and Table 19 show the calculation of residuals, for OFDM modulation at all data transfer rates that the IEEE 802.11 standard specifies. We can observe that all values match with the hypothesis H1 where all residuals differ from 0, so we are sure that these parameters cause some effect over maximum distance.

Table 20 shows the results of ANOVA for OFDM modulation.

We can see that in the case of rows, the probability value (1.3182 × 10^−8^) is lower than the one defined for α. Therefore, the variation of results is significant. On the other hand, the value of F (3.24754764) is higher than the critical value of F (1.45688671). For this reason, we discard the null hypothesis and take the alternative hypothesis. This means that the maximum distance for each frequency depend on the temperature.

On the other hand, for columns, the value of the probability (2.998 × 10^−121^) is lower than defined for α. Therefore, the variation of the results is also significant. Moreover, the value of F (153.04568) is higher than the critical value of F (1.85779125). We can discard the null hypotheses for taking the alternative which means that the maximum distance between antennae using OFDM modulation depends on the working frequency.

These results show that there are significant differences between groups which are due to the increase in the working frequency and temperature and not due to the random effect. With this, we have checked that the maximum distance for OFDM modulation are statistically different and they depend significantly on the working frequency and temperature.

Finally, there is not a clear increasing or decreasing trend in regard to maximum distances as a function of the temperatures or data transfer rates. The biggest differences are observed in the maximum distances as a function of the temperature. In addition, we can see in Figure 11, Figure 12, Figure 13 and Figure 14 that the frequency of 2412 MHz shows the greatest distance and this fact happens at 22 °C. However, the maximum distance in OFDM seems not to be affected by changes in data transfer rates and temperatures.

## 5. RTT Measurements for Each Modulation at Several Temperatures

Another important parameter to be considered on network performance is the RTT value between devices. This section presents the results of the average value of RTT registered for each test as a function of the temperature, modulation and working frequency, for maximum distances.

### 5.1. Average RTT for 16 °C

**Figure 15:**
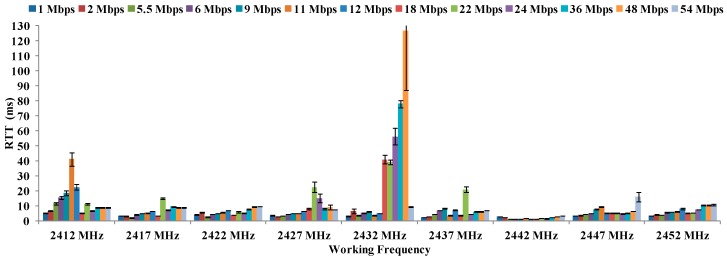
Values of RTT in ms for 16 °C.

Figure 15 shows the values of RTT (in ms) at maximum distances as a function of the working frequency for 16 °C. It is also shown the typical error for each data transfer rate. We observe that the RTT value for 11 Mbps working at 2427 MHz is about 4.85 ms. This data transfer rate presents the greatest distance value in Figure 9. Figure 14 also shows that the highest RTT value is presented for 11 Mbps at 2432 MHz. As we can see that high distances does not mean higher RTT values. At 16 °C, the best combination of settings is using OFDM modulation at 11 Mbps.

### 5.2. Average RTT for 18 °C

**Figure 16:**
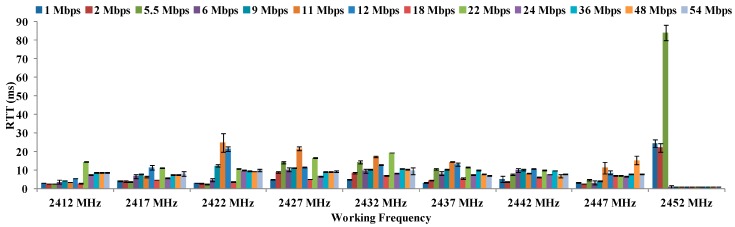
Measurements of RTT in ms for 18 °C.

Figure 16 presents the average values of RTT (in ms) depending on the working frequency at 18° C for maximum distances registered at all data transfer rates. It also presents their typical errors in ms. Figure 13 and Figure 14 showed that 11 Mbps, 12 Mbps and 18 Mbps reach the maximum distances. As Figure 16 shows, the data rate that provides lowest RTT is 18 Mbps with a value of 5.61 ms. To work at 18 °C, the best combination is to use 18 Mbps as data transfer rate, working at 2437 MHz.

### 5.3. Average RTT for 20 °C

**Figure 17:**
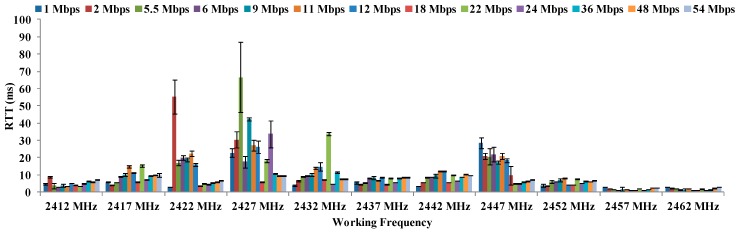
Values of RTT in ms for 20 °C.

Figure 17 shows the average values of RTT (in ms) as a function of working frequency and their typical errors for the maximum distances for all data transfer rates at 20 °C. On the one hand, we can see that 12 Mbps has a RTT value of 26.21 ms for distances of 26 cm (see Figure 13). Moreover, we can see that data rates of 1 Mbps, 2 Mbps and 5.5 Mbps (see Figure 8, Figure 9 and Figure 10) record RTT values below 9 ms for 2412 MHz, while for 2427 MHz, their values are above 22 ms. Both frequencies reach the same distances.

### 5.4. Average RTT for 22 °C

**Figure 18:**
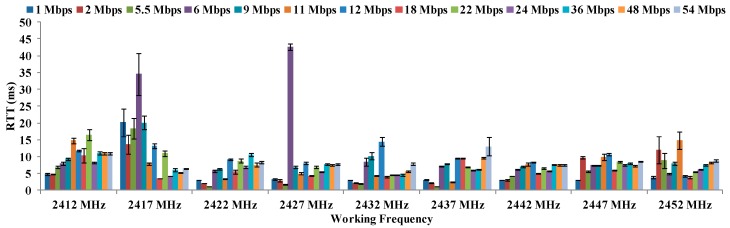
Values of RTT in ms for 22 °C.

Figure 18 shows the RTT values and their typical errors, in ms, at 22 °C for the maximum distances and all data transfer rates depending on the working frequency. On the one hand, 1 Mbps and 2 Mbps (which presents the highest distances in Figure 7 and Figure 8) present the lowest values of RTT (about 4.75 ms). Furthermore, the data rate of 22 Mbps (Figure 14) has a RTT values above 16.50 ms, which is the highest value. At 22 °C, the best combination of parameters is selecting 2412 MHz with data transfer rates of 1 Mbps and 2 Mbps.

### 5.5. Average RTT for 26 °C

**Figure 19:**
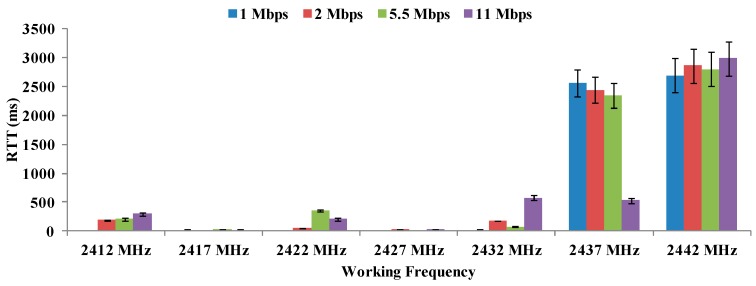
Values of RTT in ms for 26 °C.

Figure 19 shows the RTT values and their typical errors in ms as a function of working frequency for all data transfer rates at 26 °C.

As Figure 8, Figure 9 and Figure 10 showed previously, this temperature permits the lowest values of maximum distance. It this case, there are great changes in RTT values. On the one hand, frequencies of 2412 MHz and 2427 MHz (using 1 Mbps as data transfer rate) present the lowest values (about 6 ms). On the other hand, the highest frequency analyzed for this temperature, i.e., 2442 MHz presents the greatest values of RTT.

At 26 °C, the best combination of parameters would be working at 2412 MHz at 1 Mbps. However it is a situation of best combinations which offers the worst results.

As a summary of this section, we can conclude that the temperature slightly affects the value of RTT. We can observe that the lowest frequencies present smallest RTT values, but it is difficult to relate the RTT values with the network performance. This is because the line between whether an underwater network using EM waves works or not, in the case of the aquatic environment, is very subtle and therefore a gradual deterioration in terms of network performance is hardly observable.

## 6. Mathematical Model

Finally, using data shown in Section 5, it is possible to model the signal behavior as a function of the three parameters under study, i.e., temperature, modulation and working frequency. This Section is going to show these mathematical models.

The represented temperature range is from 16 to 27 °C except to CCK modulation which only shows coherent values up to 22 °C. For each modulation, we have represented all possible values which show coherent values.

The set of all our data can be represented as points of three coordinates (f, t, d), where f is frequency, t is temperature and d is the maximum distance between antennae. All points compose a surface S in the space which is the image of a continuous application (see Equation (1)):

(1) $$\vec{r}:\;D\;\subset {ℜ}^{2}\to {ℜ}^{3},$$

The set of points of the surface *S* can be represented as (see Equation (2)):

(2) $$S=\left\{\left(f,t,d\right)\in \;{ℜ}^{3}\;:\;\left(f,t,d\right)=\;\vec{r}\left(u,v\right)=f\left(u,v\right),t\left(u,v\right),d\left(u,v\right)\;\mathrm{with}\;\left(u,v\right)\in \;D\right\},$$

where u,v define the plane and $\vec{r}$ (see Equation (3)) is a continuous vectorial function defined in S, i.e.:

(3) $$\vec{r}:\;S\to {ℜ}^{3},$$

When the pair (u, v) takes all possible values, the vector $\vec{r}$ draws a surface *S* in the space ${ℜ}^{3}$. Given in mind these statements, we can define each point as the set of coordinates as follows (see Equation (4)):

(4) $$\left(f,t,d\right)=f\vec{i}+t\vec{j}+F\left(f,t\right)\vec{k},$$

Thus, the distance between antennas can be expressed as a function of the working frequency and the water temperature (see Equation (5)).

(5) d=F(f,t),

We have used Eureqa Formulize [34] to estimate the mathematical expression. Eureqa is a scientific data mining software package that searches for mathematical patterns hidden in your data. To use it, we have to import data from a *.CSV. After that, we should select the variable to be studied. The next step is to select the kind of blocks and kind of expression the program can use to make the expressions. These can be addition, subtraction, modulus, floor, etc. Finally, the program gives us a list of possible mathematical expression with the error. The last step is to select, the equations in order to check which one match with our values and is able to calculate intermediate points.

Firstly, we have used all obtained values to extract the mathematical model for underwater communications using BPSK modulation. Equation (6) relates the distance with the working frequency and the environmental temperature using BPSK modulation:

(6) $$d=669.9f+360.8t+0.393{\mathrm{ft}}^{3}-3341-8.921t^{2}-7.834{\mathrm{ft}}^{2}-0.004985{\mathrm{ft}}^{4},$$

where d represents the distance in cm, f refers to the working frequency in GHz and t is the water temperature in °C. The equation presents a correlation coefficient of 0.8676 and its average absolute error is 1.036 cm.

**Figure 20:**
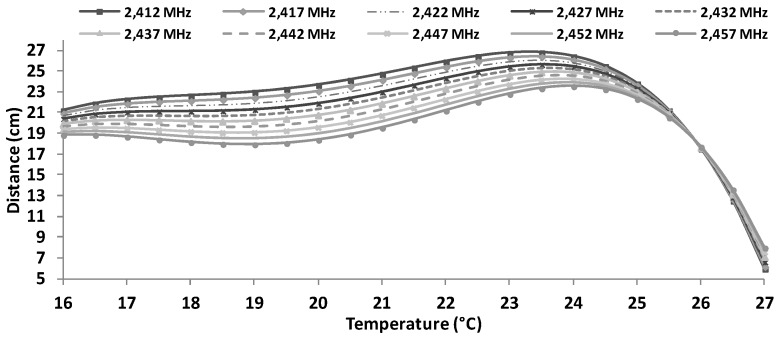
Estimated maximum distances for BPSK modulation.

Using Equation (6), it is possible to estimate the maximum distances, as a function of the working frequency and temperature, between antennae when using BPSK modulation (see Figure 20). As we can see, the maximum distance is obtained for 23.5 °C working at 2412 MHz. In addition, we can see that for temperatures of 27 °C, the maximum reached distance is about 9 cm, meanwhile for temperature around 16 °C, the maximum distances is 21 cm for 2412 MHz and less than 19 cm. for 2457 MHz.

Equation (7) relates the distance with the working frequency and the environmental temperature using QPSK modulation:

(7) $$d=594.9f^{2}+335.9t+0.3719{\mathrm{ft}}^{3}-3024-8.426t^{2}-7.336{\mathrm{ft}}^{2}-0.00475{\mathrm{ft}}^{4},$$

where d is the achieved distance in cm, f represents the frequency in MHz and t refers to the temperature in °C. Equation (7) shows a correlation coefficient of 0.8831 and an average absolute error of 0.961 cm.

**Figure 21:**
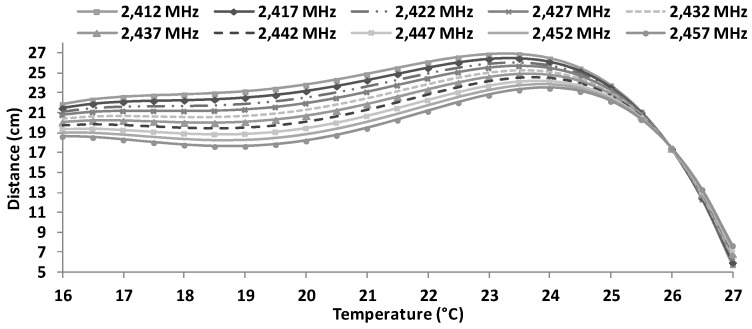
Estimated maximum distances for QPSK modulation.

Figure 21 shows, through Equation (7), the estimation of maximum distances as a function of the working frequency and temperature when the devices are using QPSK modulation. The maximum distance (26 cm) is obtained for 23.5° C working at 2412 MHz. Furthermore, we can see that for temperatures of 27 cm, the maximum reached distance is about 7 cm and for temperatures of 16 °C, we can obtain distances between 18.8 cm to 21.5 cm.

Working with OFDM, we can estimate values between 16 to 22 °C. Equation (8) relates the distance with the working frequency and the environmental temperature using QPSK modulation:

(8) $$d=226.5+0.1493t^{2}+0.3006t\times \sin\left(0.04688-t-69.33f\right)+0.1493\sin{\left(0.04688-t-69.33f\right)}^{2}+\sin\left(\tan\left(226.5-t-69.33f\right)\right)-69.33\mathrm{xf}-1.979f\left(t-\sin\left(0.04688-t-69.33f\right)\right),$$

where d is the distance in cm, f is the frequency in GHz and t the temperature in °C. Equation (8) presents a correlation coefficient of 0.9238 and its average absolute error is 0.63 cm.

**Figure 22:**
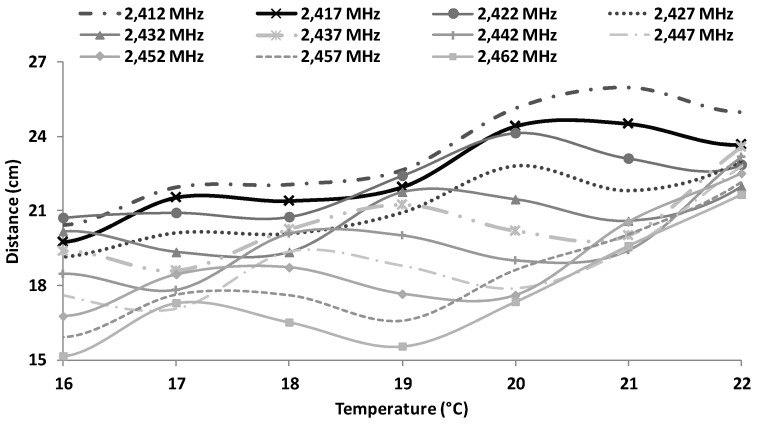
Estimated maximum distances for OFDM modulation.

Figure 22 shows the estimation of maximum distances for OFDM depending on the working frequency and temperature. These approximations are obtained from Equation (8). The maximum distance (26 cm) is obtained for 21 °C working at 2412 MHz. We can also see that for temperatures of 22 °C, the maximum reached distance is about 25 cm and for temperatures of 16 °C, we can obtain distances between 15 cm to 21 cm.

Finally, CCK transmission scheme can be modeled by Equation (9) which relates the distance with the working frequency and the environmental temperature:

(9) $$d=1538f+4.057{\mathrm{ft}}^{2}+0.003264t^{4}-2981-7.857t-0.2975t^{3}-9.28f^{3}t\;,$$

where d is the distance in cm, f is the frequency in GHz and t the temperature in °C. The correlation coefficient of Equation (9) is 0.8635 meanwhile the average absolute error is 1.16 cm.

**Figure 23:**
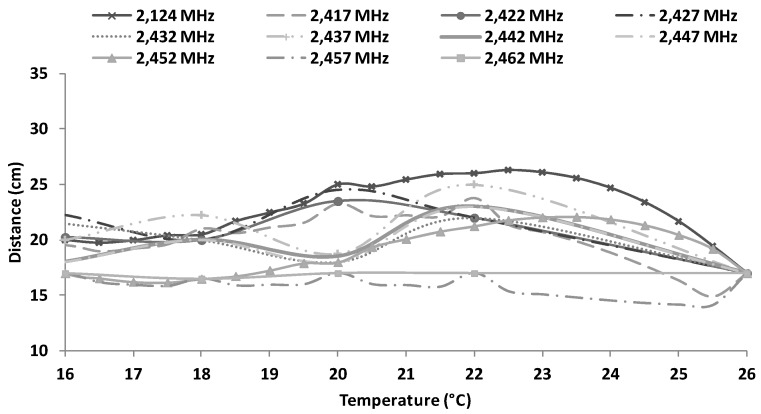
Estimated maximum distances for CCK transmission scheme.

Figure 23 shows the estimation of maximum distances for CCK transmission scheme as a function of the water temperature and working frequency. These approximations are obtained from Equation (9). The maximum distance (26 cm) is obtained for 22.5 °C working at 2412 MHz. In addition, we can see that for temperatures of 27 °C, the maximum reached distance is between 10 cm to 15 cm and for temperatures of 16 °C, we can obtain distances between 17 cm to 22 cm.

## 7. Conclusions

Underwater networks presents several application fields such as monitoring applications, as oceanographic data collection, video surveillance, pollution monitoring/detection, and off-shore oil/gas field monitoring. They can also be used for exploration applications such as submarine detection, loss treasure discovery, and hurricane disaster recovery [31].

The paper has presented the behavior of EM signals working at 2.4 GHz in fresh water underwater environments in order to evaluate if it is possible to send video through this medium. Measurements has been performed at 16, 18, 20, 22 and 26 °C and taking into account the dependence on the working frequency.

On the one hand, we have seen that it is possible to record video (with a standard resolution of 800 × 600 pixels and 30 fps) from fish farms and transmit them without errors if we have a bandwidth around 2.5 Mbps. Should one want to transmit videos with bigger resolution, we would need to increase this bandwidth. On the other hand, we have seen in practical tests about acoustic waves that the bandwidth and the data transfer rates provided by this technology are not big enough.

In addition, several previous works have claimed that EM wave propagation in fresh water has no relation with the working frequency, but our results have shown that there is an evident relationship between the maximum distance and the working frequency, type of modulation used and the water temperature. In addition, we have added a comparison of the BPSK, QPSK, CCK and OFDM schemes for several temperatures. This type of analysis has not been performed in previous studies. We have compared our results with theoretical estimations and we have checked that these values were more optimistic than those obtained on the real environment. In addition, the measurement results have shown that the water temperature affects the maximum distance we can achieve between devices. For this reason, we have modeled the value of maximum distance between underwater nodes as a parametric function that depends on the water temperature and the working frequency through which the systems transmit.

Our proposal suggests a technique for short range communications. There are several interesting applications where short range communication is suitable. Precision monitoring of ecosystems contaminated by invasive plants or hazardous waste (e.g., in swamps, the quality of the water is different depending on the season because of the water evaporation and possible uncontrolled spills that can contain harmful substances that may affect the environment and the changes of parameters such as the pH value or salinity concentration. In these situations the water is not suitable for human consumption. However, this water to supply water cooling systems for industrial uses. The neutrino telescope [35] is an underwater structure located at the bottom of the Mediterranean Sea. There is some part that needs to be wirelessly connected, such as the hydrophone and the structure. Currently, researchers use cables and penetrators to connect the different parts, but these materials are very expensive. Using our system, we could reduce the cost of this infrastructure and would avoid the critical connections that can propagate a fault (or leak) through the system.

There are other applications such as military applications, marine monitoring and even industrial applications such as marine fish farms [36] where the deployment of a UWSN would reduce the deposition of organic waste on the seabed and to enhance the marine fish farms sustainability.

The measurements presented provide several benefits. The first one is that the use of IEEE 802.11 standard is cheaper than designing and implementing a modulator and demodulator for this frequency (IEEE 802.11 devices are very cheap because of the large commercial demand). The second interesting advantage is that IEEE 802.11 provides high data transfer rates which allows the transmission of all types of sensed data, even images and video content.

As future work, we would like to extend our analysis to different kind of antennas and perform this same study in seawater. We are currently working on directional patch antennae (a single patch antenna and a patch antenna array) that will give us about 12–13 dBi. We think that with this type of antenna will reach larger distances. Thus, we will extend the experiment to these new antennae. Our aim is to apply all of these results in the aquaculture sector [36]. In addition, we want to test bigger volumes of air with distances between antenna and container proportional to integer values of lambda. In our next work we will try to define how the intrinsic features of signals, such as multipath and the intersymbol interference (ISI), affect its propagation. Finally, we want also to test the network performance for lower frequencies like 915 MHz. In this frequency, we will test the amount of video streams we can simultaneously send with enough quality.

## Figures and Tables

**Table 1:** Parameters of videos.

Frame Frequency	Parameter	Value
30 fps	Resolution	800 × 600; 852 × 480; 1024 × 768; 1280 × 720; 1280 × 1024; 1408 × 1152; 1600 × 1200; 1920 × 1080
	File format	*.mp4
	Bit Rate	Variable
60 fps	Resolution	800 × 600; 852 × 480; 1024 × 768; 1280 × 720; 1280 × 1024; 1408 × 1152; 1600 × 1200; 1920 × 1080
	File format	*.mp4
	Bit Rate	Variable

**Table 2:** Parameters analyzed in the tests performed.

Modulation	Parameter	Value
BPSK	Temperatures	16 °C, 18 °C, 20 °C, 22 °C, 26 °C
	Data transfer Rates	1 Mbps
	Frequencies tested	2412 MHz, 2417 MHz, 2422 MHz, 2427 MHz, 2432MHz, 2437 MHz, 2442 MHz, 2447 MHz, 2452 MHz, 2457 MHz, 2462 MHz
QPSK	Temperatures	16 °C, 18 °C, 20 °C, 22 °C, 26 °C
	Data transfer Rates	2 Mbps
	Frequencies tested	2412 MHz, 2417 MHz, 2422 MHz, 2427 MHz, 2432 MHz, 2437 MHz, 2442 MHz, 2447 MHz, 2452 MHz, 2457 MHz, 2462 MHz
CCK (Complementary Code Keying)	Temperatures	16 °C, 18 °C, 20 °C, 22 °C, 26 °C
	Data transfer Rates	5.5 Mbps, 11 Mbps
	Frequencies tested	2412 MHz, 2417 MHz, 2422 MHz, 2427 MHz, 2432 MHz, 2437 MHz, 2442 MHz, 2447 MHz, 2452 MHz, 2457 MHz, 2462 MHz
OFDM	Temperatures	16 °C, 18 °C, 20 °C, 22 °C
	Data transfer Rates	6 Mbps, 9 Mbps, 12 Mbps, 18 Mbps, 22 Mbps, 24 Mbps, 36 Mbps, 48 Mbps, 54 Mbps
	Frequencies tested	2412 MHz, 2417 MHz, 2422 MHz, 2427 MHz, 2432 MHz, 2437 MHz, 2442 MHz, 2447 MHz, 2452 MHz, 2457 MHz, 2462 MHz

**Table 3:** Mean values to determine whether factors has some effect over the variable to be analyzed.

		Factor 2 (β)				
	Levels	1	2	…	J	Mean per Row
**Factor 1 (α)**	1	Y_11_	Y_12_	…	Y_1J_	${\bar{Y}}_{1.}$
	2	Y_21_	Y_22_	…	Y_2J_	${\bar{Y}}_{2.}$
	…	…	…	…	…	…
	I	Y_I1_	Y_I2_	…	Y_IJ_	${\bar{Y}}_{I.}$
	Mean per Column	${\bar{Y}}_{.1}$	${\bar{Y}}_{.2}$	...	${\bar{Y}}_{.J}$	${\bar{Y}}_{..}$

**Table 4:** Calculation of residuals for BPSK.

Data Transfer Rates	2412 MHz	2417 MHz	2422 MHz	2427 MHz	2432 MHz	2437 MHz	2442 MHz	2447 MHz	2452 MHz	2457 MHz	2462 MHz
1 Mbps at 16 °C	2.50	1.80	0.70	0.80	2.20	1.10	1.30	1.75	2.00	-	-
1 Mbps at 18 °C	1.00	0.30	0.80	1.20	0.20	1.10	0.70	0.25	0.01	-	-
1 Mbps at 20 °C	3.50	2.70	2.70	3.80	1.80	1.40	0.80	1.75	1.00	0.1	0.2
1 Mbps at 22 °C	4.50	3.70	1.20	0.80	2.20	2.10	3.70	3.25	3.00	-	-
1 Mbps at 26 °C	4.50	4.30	3.80	4.20	2.80	2.90	2.30	-	-	-	-

**Table 5:** ANOVA for BPSK.

Origin of Variations	Sum of Squares	Degree of Freedom	Average Squares	F	Probability	Critical Value for F
Temperatures	568.154545	4	142.038636	7.60026754	0.00011845	2.60597495
Frequency	2321.54545	10	232.154545	12.4222303	1.9998 × 10^−9^	2.07724805
Error	747.545455	40	18.6886364			
Total	3637.24545	54				

**Table 6:** Calculation of residuals for QPSK.

Data Transfer Rates	2412 MHz	2417 MHz	2422 MHz	2427 MHz	2432 MHz	2437 MHz	2442 MHz	2447 MHz	2452 MHz	2457 MHz	2462 MHz
2 Mbps at 16 °C	1.7	1.7	0.7	0.8	2.2	0.5	1.3	1.2	1.375	-	-
2 Mbps at 18 °C	1.2	0.2	0.8	1.2	0.2	0.5	0.7	0.8	1.375	-	-
2 Mbps at 20 °C	3.3	2.3	2.7	3.8	1.8	2	0.8	1.2	0.375	0.1	0.15
2 Mbps at 22 °C	4.3	3.8	1.2	0.8	2.2	4.5	3.7	3.8	3.125	-	-
2 Mbps at 26 °C	4.7	4.2	3.8	4.2	2.8	3.5	2.3	2.2	-	-	-

**Table 7:** ANOVA for QPSK.

Origin of Variations	Sum of Squares	Degree of Freedom	Average Squares	F	Probability	Critical Value for F
Temperatures	403.3	4	100.825	6.71606994	0.00031136	2.60597495
Frequency	2384.72727	10	238.472727	15.8849444	5.3453 × 10^−11^	2.07724805
Error	600.5	40	15.0125			
Total	3388.52727	54				

**Table 8:** Calculation of residuals for CCK at 5.5 Mbps.

Data Transfer Rates	2412 MHz	2417 MHz	2422 MHz	2427 MHz	2432 MHz	2437 MHz	2442 MHz	2447 MHz	2452 MHz	2457 MHz	2462 MHz
5.5 Mbps at 16 °C	1.7	1.7	0.7	0.8	2.2	0.5	1.3	1.2	1.375	-	-
5.5 Mbps at 18 °C	1.2	0.2	0.8	1.2	0.2	0.5	0.7	0.8	1.375	-	-
5.5 Mbps at 20 °C	3.3	2.3	2.7	3.8	1.8	2	0.8	1.2	0.375	0.12	0.05
5.5 Mbps at 22 °C	4.3	3.8	1.2	0.8	2.2	4.5	3.7	3.8	3.125	-	-
5.5 Mbps at 26 °C	4.7	4.2	3.8	4.2	2.8	3.5	2.3	2.2	-	-	-

**Table 9:** Calculation of residuals for CCK at 11 Mbps.

Data Transfer Rates	2412 MHz	2417 MHz	2422 MHz	2427 MHz	2432 MHz	2437 MHz	2442 MHz	2447 MHz	2452 MHz	2457 MHz	2462 MHz
11 Mbps at 16 °C	1.7	1.1	0.1	1.4	1.4	1.5	1.3	1.75	1	-	-
11 Mbps at 18 °C	1.2	0.4	0.6	1.1	0.4	2	0.7	0.25	2	-	-
11 Mbps at 20 °C	3.3	2.4	2.9	2.9	1.6	1.5	0.8	1.75	0.07	0.2	3.3
11 Mbps at 22 °C	4.3	1.9	1.4	0.9	2.4	4.5	3.7	3.25	3	-	-
11 Mbps at 26 °C	4.7	3.6	3.6	4.1	2.6	3.5	2.3	-	-	-	-

**Table 10:** ANOVA for CCK.

Origin of Variations	Sum of Squares	Degree of Freedom	Average Squares	F	Probability	Critical Value for F
Temperatures	1134.11136	9	126.012374	7.79263227	2.0984 × 10^−8^	1.98559496
Frequency	4668.09091	10	466.809091	28.8675745	6.1007 × 10^−24^	1.93756679
Error	1455.36364	90	16.1707071			
Total	7257.56591	109				

**Table 11:** Calculation of residuals for OFDM at 6 Mbps.

Data Transfer Rates	2412 MHz	2417 MHz	2422 MHz	2427 MHz	2432 MHz	2437 MHz	2442 MHz	2447 MHz	2452 MHz	2457 MHz	2462 MHz
6 Mbps at 16 °C	2.875	2.625	1	0.01	1.5	0.625	1.875	1.625	1.25	-	-
6 Mbps at 18 °C	2.375	1.125	1.5	2	0.5	0.375	0.125	0.125	1.25	-	-
6 Mbps at 20 °C	2.125	0.875	2	2	2.5	3.125	1.375	1.625	0.25	0.03	0.22
6 Mbps at 22 °C	3.125	2.875	0.5	0.02	1.5	3.375	3.125	3.375	2.75	-	-

**Table 12:** Calculation of residuals for OFDM at 9 Mbps.

Data Transfer Rates	2412 MHz	2417 MHz	2422 MHz	2427 MHz	2432 MHz	2437 MHz	2442 MHz	2447 MHz	2452 MHz	2457 MHz	2462 MHz
9 Mbps at 16 °C	2.875	2.5	0.875	0.02	1.5	0.625	1.875	1.625	1	-	-
9 Mbps at 18 °C	2.375	1	1.375	2	0.5	0.375	0.125	0.125	2	-	-
9 Mbps at 20 °C	2.125	1.5	1.625	2	2.5	3.125	1.375	1.625	0.03	0.06	0.03
9 Mbps at 22 °C	3.125	2	0.625	0.03	1.5	3.375	3.125	3.375	3	-	-

**Table 13:** Calculation of residuals for OFDM at 12 Mbps.

Data Transfer Rates	2412 MHz	2417 MHz	2422 MHz	2427 MHz	2432 MHz	2437 MHz	2442 MHz	2447 MHz	2452 MHz	2457 MHz	2462 MHz
12 Mbps at 16 °C	2.875	2.375	1	0.5	0.75	2.375	1.5	1.375	1.5	-	-
12 Mbps at 18 °C	2.375	0.375	1.5	2.5	0.25	1.125	0	0.125	2.5	-	-
12 Mbps at 20 °C	2.125	1.125	2	3.5	2.25	2.375	1.5	1.875	0.5	0.5	0.08
12 Mbps at 22 °C	3.125	1.625	0.5	0.5	1.75	3.625	3	3.125	4.5	-	-

**Table 14:** Calculation of residuals for OFDM at 18 Mbps.

Data Transfer Rates	2412 MHz	2417 MHz	2422 MHz	2427 MHz	2432 MHz	2437 MHz	2442 MHz	2447 MHz	2452 MHz	2457 MHz	2462 MHz
18 Mbps at 16 °C	2.875	2.125	1	0.5	0.75	2.375	1.5	1.25	1.5	-	-
18 Mbps at 18 °C	2.375	0.125	1.5	2	0.25	1.125	0	0.25	2.5	-	-
18 Mbps at 20 °C	2.125	1.375	2	2	2.25	2.375	1.5	1.75	0.5	0.03	0.01
18 Mbps at 22 °C	3.125	0.875	0.5	0.01	1.75	3.625	3	3.25	4.5	-	-

**Table 15:** Calculation of residuals for OFDM at 22 Mbps.

Data Transfer Rates	2412 MHz	2417 MHz	2422 MHz	2427 MHz	2432 MHz	2437 MHz	2442 MHz	2447 MHz	2452 MHz	2457 MHz	2462 MHz
22 Mbps at 16 °C	3	2	1	0.07	0.5	2	1.5	1.25	1.5	-	-
22 Mbps at 18 °C	2	0.5	1.5	2	1.5	0.1	0.09	0.25	2.5	-	-
22 Mbps at 20 °C	2	1	2	2	0.5	2	1.5	1.75	0.5	0.02	0.01
22 Mbps at 22 °C	3	1.5	0.5	0.2	0.5	4	3	3.25	4.5	--	

**Table 16:** Calculation of residuals for OFDM at 24 Mbps.

Data Transfer Rates	2412 MHz	2417 MHz	2422 MHz	2427 MHz	2432 MHz	2437 MHz	2442 MHz	2447 MHz	2452 MHz	2457 MHz	2462 MHz
24 Mbps at 16 °C	3	2	1	0.09	0.5	2	1.5	1.25	1.5		
24 Mbps at 18 °C	2	0.5	1.5	2	1.5	0.05	0.04	0.25	2.5		
24 Mbps at 20 °C	2	1	2	2	0.5	2	1.5	1.75	0.5	0.1	0.2
24 Mbps at 22 °C	3	1.5	0.5	0.1	0.5	4	3	3.25	4.5	-	-

**Table 17:** Calculation of residuals for OFDM at 36 Mbps.

Data Transfer Rates	2412 MHz	2417 MHz	2422 MHz	2427 MHz	2432 MHz	2437 MHz	2442 MHz	2447 MHz	2452 MHz	2457 MHz	2462 MHz
36 Mbps at 16 °C	3	2	0.25	0.1	0.125	2	1.5	1.25	1.5	-	-
36 Mbps at 18 °C	2	0.5	1.75	2	2.375	0.13	0.08	0.25	2.5	-	-
36 Mbps at 20 °C	2	1	1.75	2	2.125	2	1.5	1.75	0.5	0.01	0.02
36 Mbps at 22 °C	3	1.5	0.25	0.12	0.125	4	3	3.25	4.5	-	-

**Table 18:** Calculation of residuals for OFDM at 48 Mbps.

Data Transfer Rates	2412 MHz	2417 MHz	2422 MHz	2427 MHz	2432 MHz	2437 MHz	2442 MHz	2447 MHz	2452 MHz	2457 MHz	2462 MHz
48 Mbps at 16 °C	3	2	0.125	0.01	0.25	1.875	1.625	1	1.25	-	-
48 Mbps at 18 °C	2	0.5	1.625	2	1.25	0.125	0.125	0.03	2.25	-	-
48 Mbps at 20 °C	2	1	1.375	2	1.75	1.875	1.125	2	1.25	0.01	0.02
48 Mbps at 22 °C	3	1.5	0.375	0.02	0.25	3.625	2.875	3	4.75	-	-

**Table 19:** Calculation of residuals for OFDM at 54 Mbps.

Data Transfer Rates	2412 MHz	2417 MHz	2422 MHz	2427 MHz	2432 MHz	2437 MHz	2442 MHz	2447 MHz	2452 MHz	2457 MHz	2462 MHz
54 Mbps at 16 °C	3	2.375	0.125	0.03	0.25	2	1.625	1.125	1.25	-	-
54 Mbps at 18 °C	2	0.875	1.625	2	1.25	0.02	0.125	0.375	2.25	-	-
54 Mbps at 20 °C	2	0.625	1.375	2	1.75	2	1.125	2.125	1.25	0.01	0.02
54 Mbps at 22 °C	3	2.625	0.375	0.01	0.25	4	2.875	2.875	4.75	-	-

**Table 20:** ANOVA for OFDM.

Origin of variations	Sum of squares	Degree of Freedom	Average Squares	F	Probability	Critical Value for F
Temperatures	1270.41604	35	36.297601	3.24754764	1.3182 × 10^−8^	1.45688671
Frequency	17105.803	10	1710.5803	153.04568	2.998 × 10^−121^	1.85779125
Error	3911.92424	350	11.1769264			
Total	22288.1433	395				
